# Tinea corporis infection manifestating as retinochoroiditis—an unusual presentation

**DOI:** 10.1186/s12348-019-0173-z

**Published:** 2019-05-27

**Authors:** Manisha Agarwal, Chanda Gupta, Gaganjeet Singh Gujral, Mamta Mittal

**Affiliations:** 1 Dr Shroff’s Charity Eye Hospital, 5072, Kedarnath Road, Daryaganj, New Delhi, 110002 India; 2 Consultant Dermatologist, Agra, India

## Abstract

**Background:**

*Tinea corporis*, a superficial dermatophyte, is a fungal infection of the body. Ocular involvement due to dermatophytes can present as eyelid infestation. Various cases of retinochoroiditis have been reported secondary to infective etiology such as *Toxoplasma gondii*, *Candida albicans*, *Trichosporon beigelii*, and *Sporotrichum schenkii*. However, retinochoroiditis secondary to fungal infection of the skin caused by *T. corporis* has not been reported in the past.

**Findings:**

A 45-year-old female presented with blurring of vision in the left eye for the last 20 days with a history of very severe itching on the abdomen and back. She had been diagnosed to have *T. corporis* infection by a dermatologist in the past, however, was non-compliant with the treatment. Anterior segment was within normal limits. Fundus examination of the right eye was normal and left eye showed a diffuse yellowish retinochoroiditis patch with irregular margins at the inferotemporal arcade. Optical coherence tomography (OCT) of the left eye through the macula showed shallow subretinal fluid with hyperreflective dots and passing through the retinochoroitidis patch showed increased retinal thickening with a pigment epithelial detachment and subretinal fluid. Left eye fundus fluorescein angiography (FFA) showed three hyperfluorescent areas along the inferotemporal arcade increasing in size and intensity with blurring of margins in the late phases. She had extensive reddish color erythematous plaque-like skin lesions over the abdomen and back. Treatment with oral itraconazole resulted in complete resolution of retinochoroiditis. Itraconazole is an orally active, triazole anti-fungal agent found to be effective in the management of dermatomycosis.

**Conclusion:**

We report this case to highlight that one must rule out an infective etiology of retinochoroiditis before starting oral corticosteroids as it may worsen the infection especially fungal as in our patient. A detailed medical history and thorough examination helped us in diagnosing a systemic infective pathology and the possible cause of retinochoroiditis. To the best of our knowledge, this is the first case of infective retinochoroiditis secondary to *T. corporis* to be reported.

## Introduction

Dermatophytes are superficial fungi that infect and multiply within keratinized tissues. *Tinea corporis* is known to affect the body and mostly present as erythematous scaly papules that gradually progress to annular or nummular red patches or plaques, frequently with central clearing and peripheral scales. [1, 2] Ocular involvement due to dermatophytes can present as eyelid infestation. [3] We report a case of retinochoroiditis secondary to fungal infection of the skin due to *Tinea corporis*.

## Case report

**Fig. 1:**
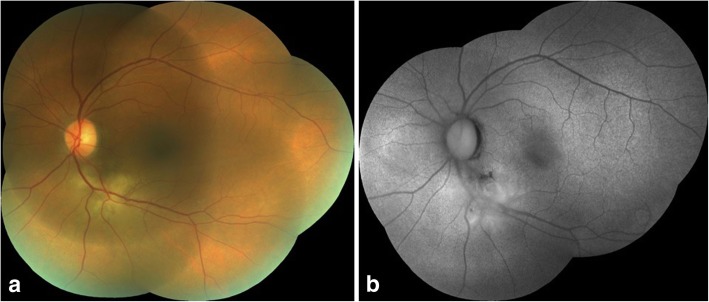
**a** Fundus photograph of the left eye showing a diffuse yellowish retinochoroiditis patch with irregular margins along the inferotemporal arcade. **b** FAF photograph of the left eye showing an ill-defined area of hyperautofluorescence along the inferotemporal arcade

**Fig. 2:**
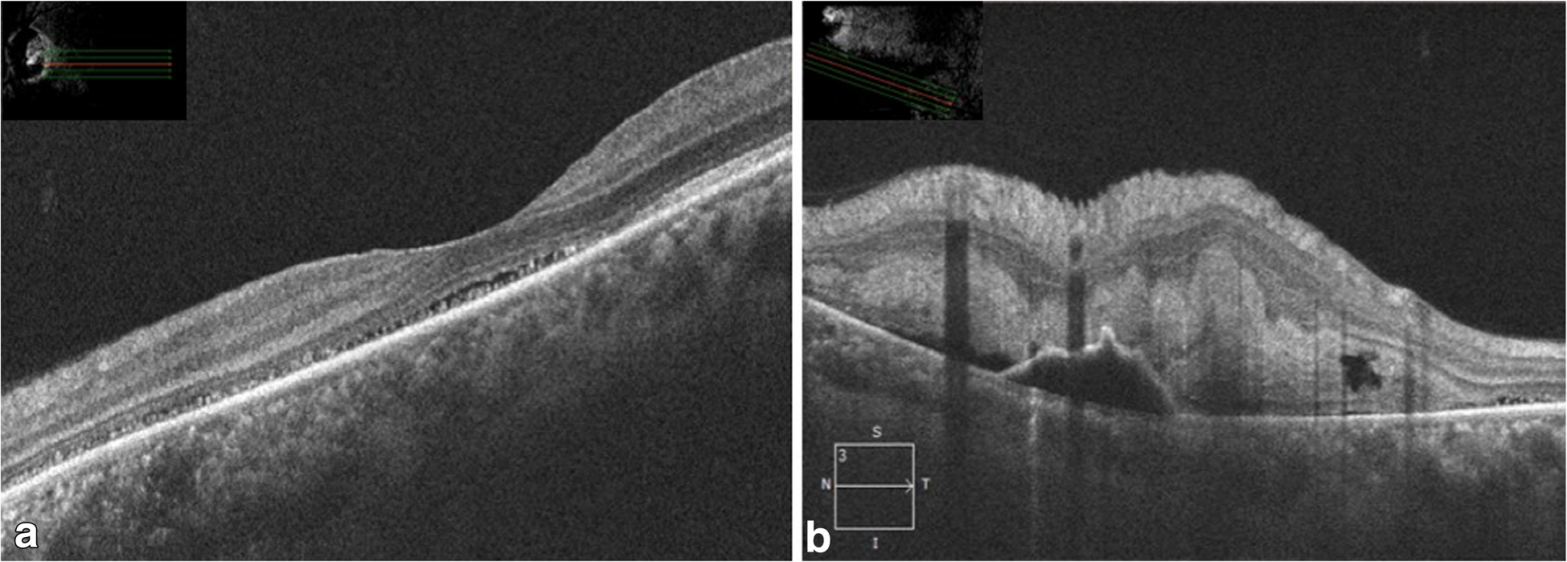
**a** OCT scan of the left eye showing normal foveal contour with subretinal fluid and high reflective dots. **b** OCT scan through the retinochoroiditis lesion showing increased retinal thickening with a pigment epithelial detachment and subretinal fluid

**Fig. 3:**
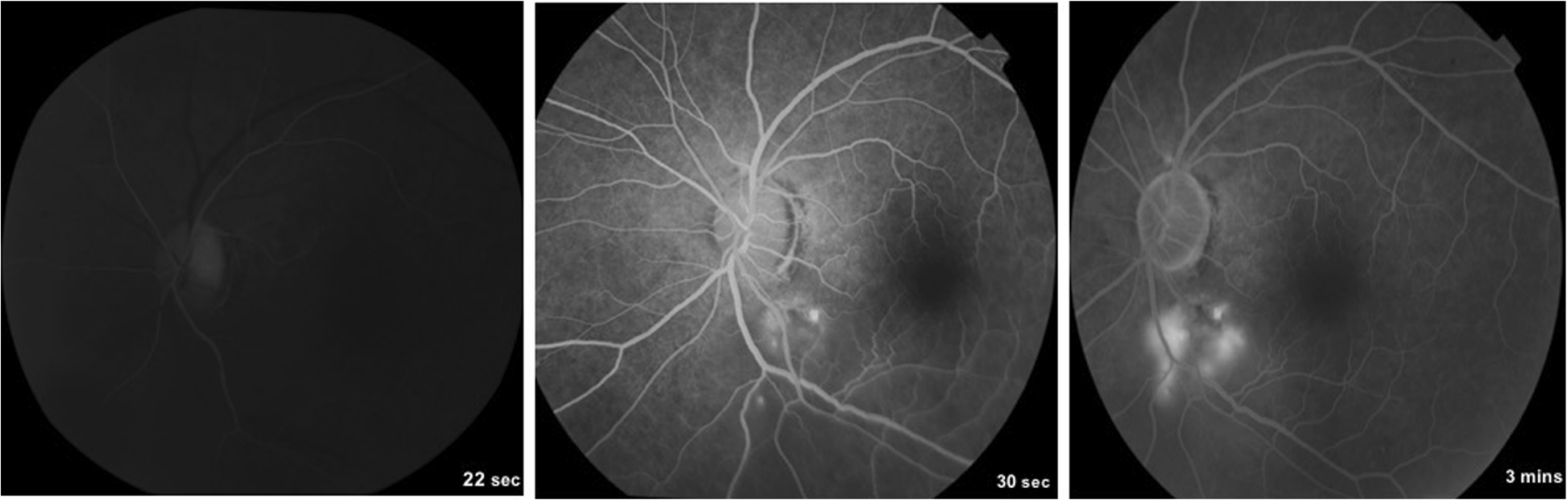
**a** Left eye FFA showing three hyperfluorescent areas along the inferotemporal arcade increasing in size and intensity with blurring of margins in the late phases. (sec, seconds; min, minutes)

A 45-year-old female presented with complaints of blurring of vision in the left eye for the last 20 days with a history of very severe itching on the abdomen and back. She was a known case of hypertension on treatment for the last 2 years. She had been diagnosed to have *T. corporis* infection by a dermatologist in the past, however, was non-compliant with the treatment. There was no history of intake of steroids in any form. Best corrected visual acuity (BCVA) was 6/6, N6 in the right eye and 6/9, N8 in the left eye. Applanation tonometry recorded intraocular pressures of 19 mmHg and 18 mmHg respectively. Anterior segment was within normal limits. Fundus examination of the right eye was normal and the left eye examination showed a diffuse yellowish retinochoroiditis patch with irregular margins at the inferotemporal arcade [Fig. 1a]. Fundus autofluorescence (FAF) of the left eye also showed an ill-defined area of hyperautofluorescence along the inferotemporal arcade. [Fig. 1b]. OCT of the left eye through the macula showed shallow subretinal fluid with hyperreflective dots and passing through the retinochoroitidis patch showed increased retinal thickening with a pigment epithelial detachment and subretinal fluid. [Fig. 2a, b]. Left eye fundus fluorescein angiography showed three hyperfluorescent areas along the inferotemporal arcade increasing in size and intensity with blurring of margins in the late phases [Fig. 3].

**Fig. 4:**
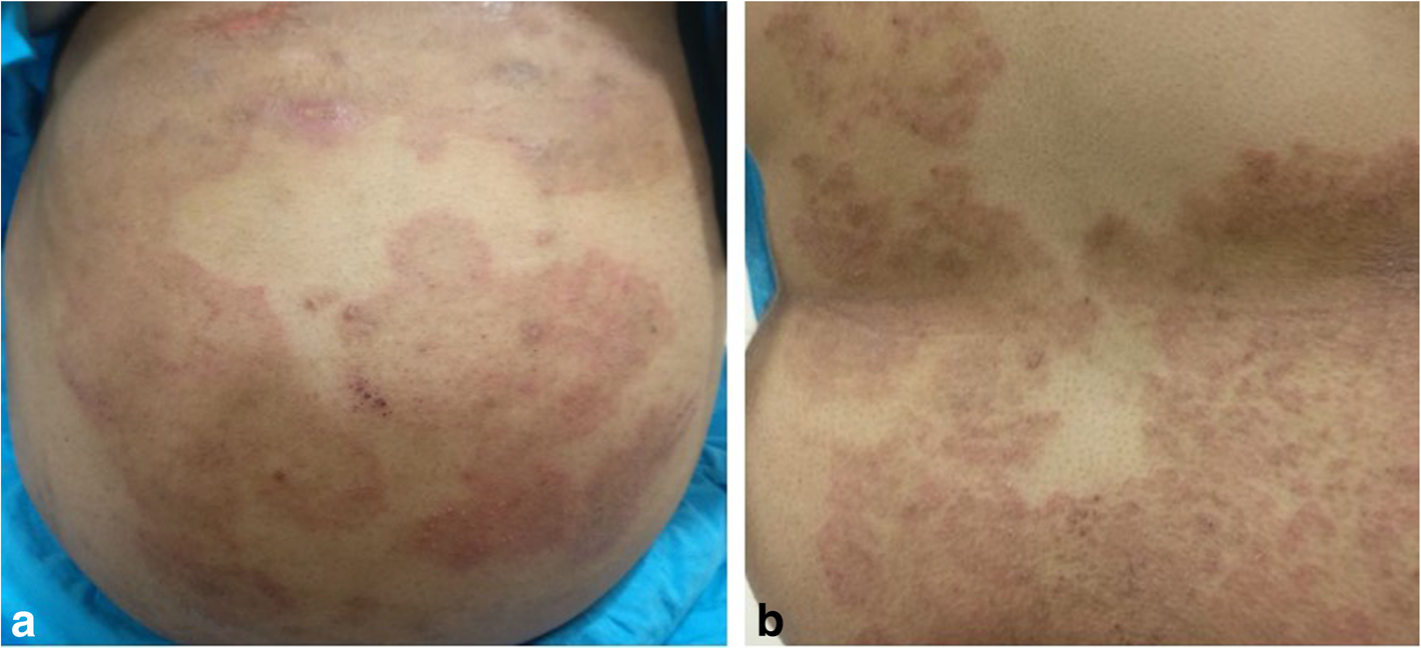
**a**, **b** Color photograph of the abdomen and back showing extensive reddish color erythematous plaque-like skin lesions

On general examination, she had extensive reddish color erythematous plaque-like skin lesions over the abdomen and back (Fig. 4a, b). Hematological investigations showed hemoglobin 11 g/dl, total leucocyte count 9600 cells/cu mm, differential leucocyte count showed increased eosinophils to 12, absolute eosinophil count was raised to 1150 cells/cu mm, ESR was raised to 50 mm first hour, kidney and thyroid function tests were normal, urine examination showed increased pus cells and epithelial cells, the Mantoux test for tuberculosis was negative, and Treponema pallidum hemagglutination assay (TPHA) was negative for syphilis. She was sent for a dermatologist’s opinion who diagnosed her with *T. corporis* infection and started her on oral itraconazole 200 mg twice a day for 2 weeks followed by once a day for 1 month and Elovera lotion for local application on skin lesions.

**Fig. 5:**
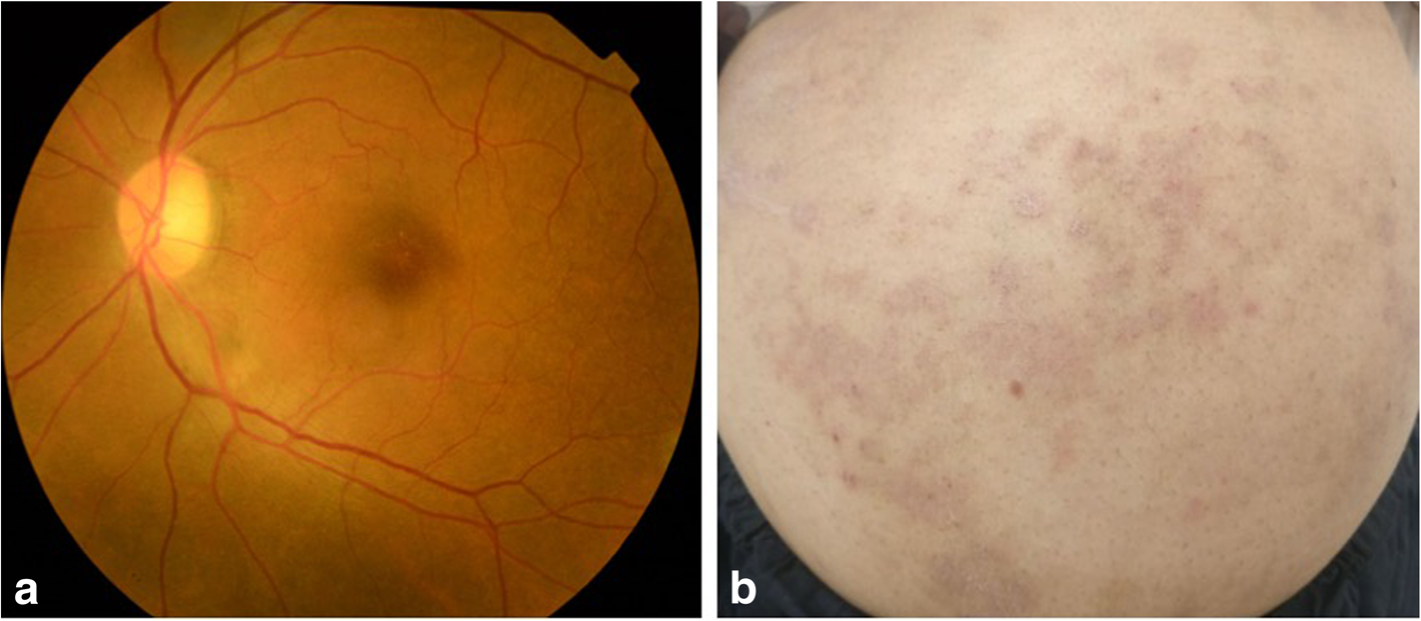
**a** Fundus photograph of the left eye showing resolving retinochoroiditis lesion. **b** Color photograph of the abdomen showing resolving skin lesions. (Second week)

**Fig. 6:**
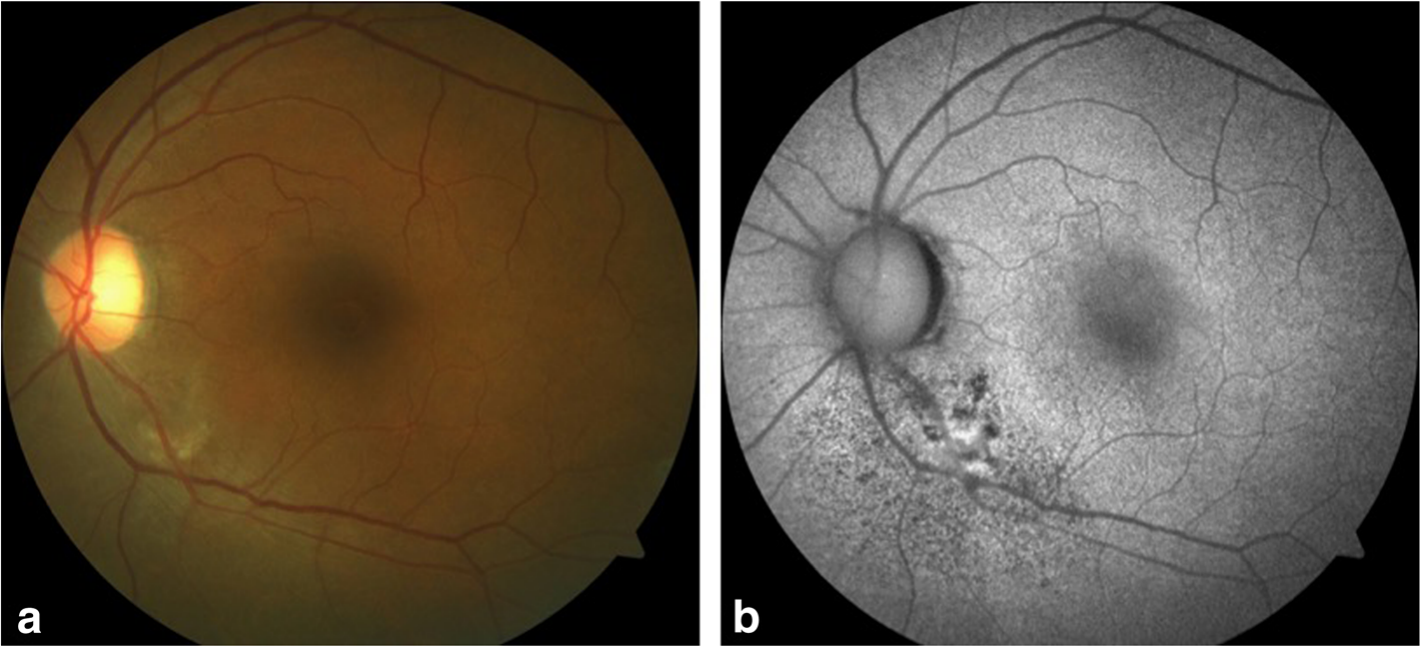
**a** Fundus photograph of the left eye showing healed retinochoroiditis lesion with chorioretinal atrophy patches. **b** Fundus autofluorescence of the left eye showing hypoautofluorescent spots surrounding the predominantly hyperautofluorescent lesion. (12th week)

**Fig. 7:**
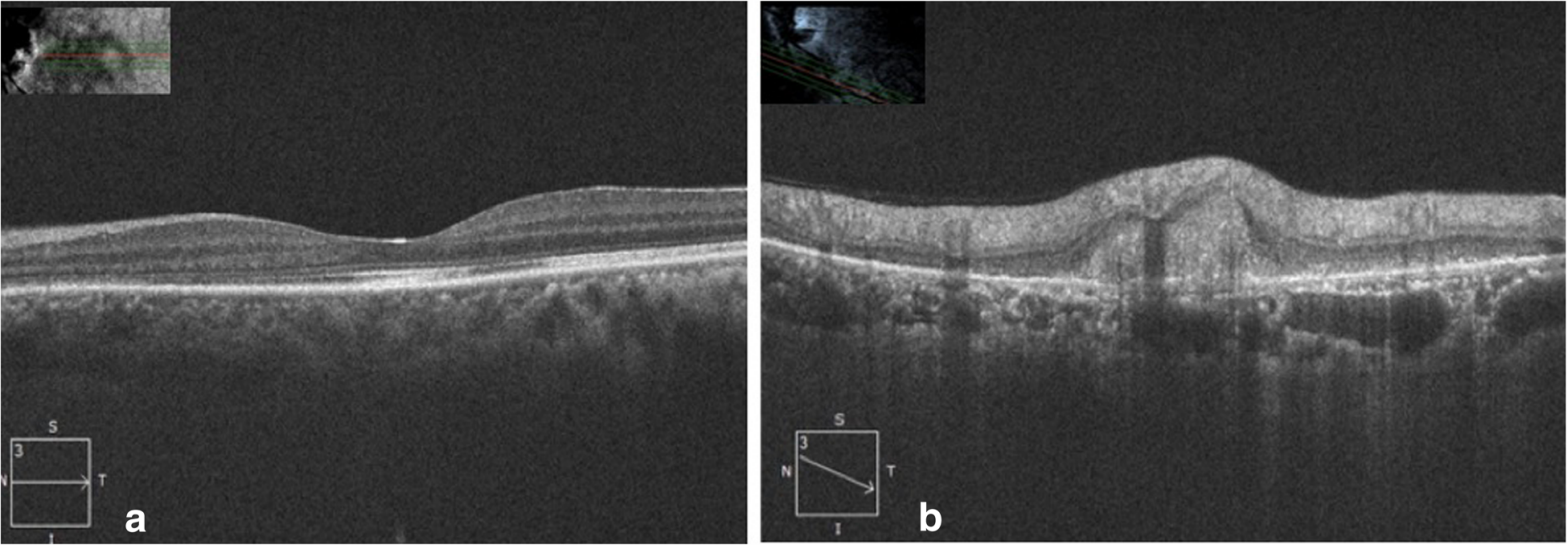
**a** OCT of the left eye through the macula showing resolved subretinal fluid. **b** OCT of the left eye through the retinochoroiditis lesion showing decreased retinal thickening with resolved PED and subretinal fluid

On follow-up at 2 weeks, she was symptomatically better, and retinochoroidal lesion had started healing and skin lesions improved drastically [Fig. 5a, b]. A similar outcome was noted at third and eighth week of follow-ups. At 12th week, healed patches of choroidal lesions were seen and she improved to 6/6, N6 in the left eye [Fig. 6a]. FAF of the left eye did not show any active lesions [Fig. 6b]. OCT scan of the left eye through the macula showed resolved subretinal fluid and through the retinochoroiditis lesion showed decreased retinal thickening with resolved PED and subretinal fluid [Fig. 7a, b].

## Discussion

Dermatophytes are superficial fungi that infect and multiply within keratinized tissues. Tinea corporis is known to affect the body [1]. Ocular involvement due to dermatophytes can present as eyelid infestation [2]. Diagnosis of *T. corporis* is mainly made on clinical examination and can be confirmed by microscopy and culture of skin scrapings, histopathology, and polymerase chain reaction [4]. On review of literature, cases of retinochoroiditis have been reported secondary to infective etiology such as ocular toxoplasmosis presenting as retinal vasculitis with focus of retinochoroiditis as pigmented central area [5]; fungal infections such as *Candida albicans* as central subretinal neovascularisation [6], Trichosporon chorioretinitis as an elevated choroidal lesion with subretinal hemorrhage [7], and necrotizing granulomatous retinochoroiditis due to *Sporotrichum schenkii* [8]. However, retinochoroiditis secondary to fungal infection of the skin caused by *T. corporis* has not been reported in the past.

Our patient had undergone skin biopsy and diagnosed to have *T. corporis* infection in the past however was non-compliant with the treatment. After the development of retinochoroiditis, she was again referred to a dermatologist who diagnosed it as *T. corporis* infection and started her on oral anti-fungal medications. No active intervention was done for retinochoroiditis except the patient was kept under close follow-up at every visit, to ensure that there was no extension threatening the macula. To our surprise, there was a complete resolution of retinochoroiditis after 12 weeks of oral itraconazole therapy. This therapeutic response confirmed that the retinochoroiditis was secondary to *T. corporis* infection. Itraconazole is an orally active, triazole anti-fungal agent found to be effective in the management of *T. corporis* [9, 10].

We report this case to highlight that one must rule out an infective etiology of retinochoroiditis before starting oral corticosteroids as it may worsen the infection especially fungal as in our patient. A detailed medical history and thorough examination helped us in diagnosing a systemic infective pathology and the possible cause of retinochoroiditis. To the best of our knowledge, there is no case of infective retinochoroiditis secondary to *T. corporis* by PUBMED search and ours is the first case to be reported.

## Abbreviations

BCVA: Best corrected visual acuity
FAF: Fundus autofluorescence
FFA: Fundus fluorescein angiography
OCT: Optical coherence tomography
PED: Pigment epithelial detachment

## Author’s information

Dr. Shroff’s Charity Eye Hospital, 5027, Kedarnath Road, Daryaganj, New Delhi 110,002, India.
